# Supplementary material for: The genetic risk for hypertension is lower among the Hungarian Roma population compared to the general population
Source: PLoS One. 2020 Jun 17;15(6):e0234547. doi: 10.1371/journal.pone.0234547 (PMC7299387; doi:10.1371/journal.pone.0234547)
Supplement: S1 File — (PDF) [file pone.0234547.s001.pdf]

Supporting Information

The genetic risk for hypertension is lower among the Hungarian Roma population compared to the general population

Beáta Soltész[1], Péter Pikó[3], János Sándor[2,4], Zsigmond Kósa[5], Róza Ádány[2,3,4], Szilvia Fiatal[2,4]

[1] Doctoral School of Health Sciences, Department of Preventive Medicine, Faculty of Public Health, University of Debrecen, Debrecen, Hungary

[2] Department of Preventive Medicine, Faculty of Public Health, University of Debrecen, Debrecen, Hungary

[3] MTA-DE Public Health Research Group of the Hungarian Academy of Sciences, Faculty of Public Health, University of Debrecen, Debrecen, Hungary

[4] WHO Collaborating Centre on Vulnerability and Health, Department of Preventive Medicine, Faculty of Public Health, University of Debrecen, Debrecen, Hungary

[5] Department of Health Visitor Methodology and Public Health, Faculty of Health, University of Debrecen, Nyíregyháza, Hungary

Supplementary Figure 1. Details of the systematic review search according to the PRISMA Statement

<u>A) The keywords were used for the literature search:</u>

PubMed:

(„essential hypertension" OR „blood pressure") AND

(„molecular genetics" OR „genomics" OR „genes") AND

(„single-nucleotide polymorphism" OR „genetic variants" OR „gene polymorphism" OR „common gene variants") AND

(„genome-wide association study" OR „GWAS" OR „candidate gene study" OR „case-control study" OR „meta-analysis" OR „review" OR „association")

filters: Humans, English

time frame: 11/30/2015 or earlier

Huge Navigator/Literature Finder:

(„essential hypertension" OR „blood pressure") AND

(„molecular genetics" OR „genomics" OR „genes") AND

(„single-nucleotide polymorphism" OR „genetic variants" OR „gene polymorphism" OR „common gene variants") AND

(„genome-wide association study" OR „GWAS" OR „candidate gene study" OR „case-control study" OR „meta-analysis" OR „review" OR „association")

filters: disease-->hypertension

time frame: 11/30/2015 or earlier

NHGRI-EBI GWAS Catalog

filter: disease-->hypertension

time frame: 11/30/2015 or earlier

B) The flow-chart of the systematic search according to the PRISMA Statement

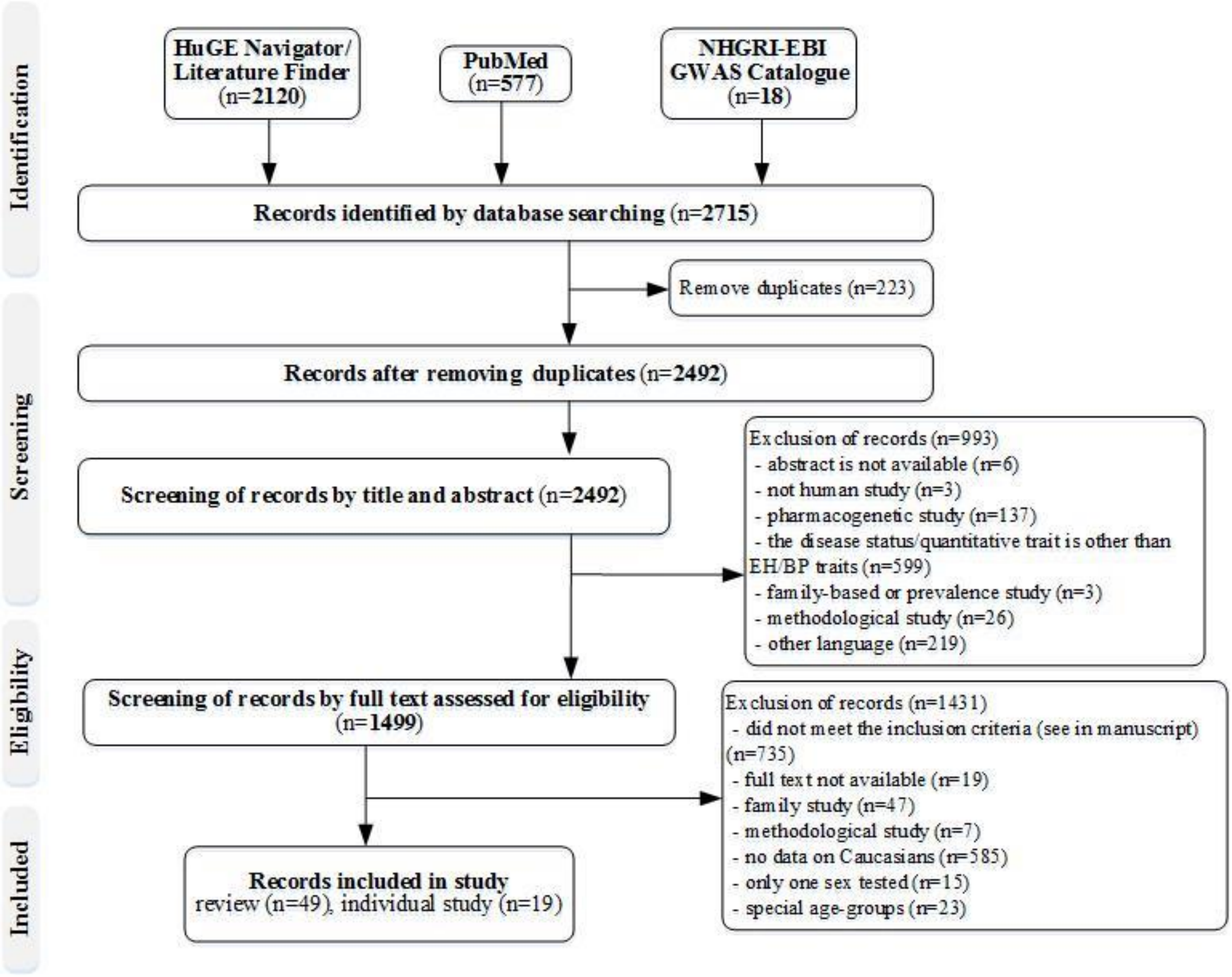

From: Moher D, Liberati A, Tetzlaff J, Altman DG, The PRISMA Group (2009). *P*referred *R*eporting *I*tems for *S*ystematic Reviews and *M*eta-*A*nalyses: The PRISMA Statement. PLoS Med 6(7): e1000097. doi:10.1371/journal.pmed1000097

Supplementary Table 1. Selection process of the SNPs included in the study

Step 1.

SNPs chosen after literature review				
Number	SNP	Locus	Gene name(s)	Reference
1	rs4305	*ACE*	Angiotensin I converting enzyme	[1]
2	rs4341	*ACE*	Angiotensin I converting enzyme	[2] [3]
3	rs4961	*ADD1*	Adducin 1	[4] [5]
4	rs1801253	*ADRB1*	Adrenoceptor beta 1	[1]
5	rs2004776	*AGT*	Angiotensinogen	[1] [6] [7] [8]
6	rs699	*AGT*	Angiotensinogen	[9] [10]
7	rs4762	*AGT*	Angiotensinogen	[10] [11]
8	rs5049	*AGT*	Angiotensinogen	[11]
9	rs5186	*AGTR1*	Angiotensin II receptor type 1	[12]
10	rs17249754	*ATP2B1*	ATPase plasma membrane Ca2+ transporting 1	[8] [13]
11	rs2681472	*ATP2B1*	ATPase plasma membrane Ca2+ transporting 1	[8] [14]
12	rs4590817	*C10orf107*	Chromosome 10 open reading frame 107	[13]
13	rs11014166	*CACNB2*	Calcium voltage-gated channel auxiliary subunit beta 2	[7] [8] [14]
14	rs1813353	*CACNB2(3')*	Calcium voltage-gated channel auxiliary subunit beta 2	[8] [13]
15	rs4373814	*CACNB2(5')*	Calcium voltage-gated channel auxiliary subunit beta 2	[13]
16	rs1799998	*CYP11B2*	Cytochrome P450 family 11 subfamily B member 2	[15]
17	rs1378942	*CYP1A1-ULK3*	Cytochrome P450 family 1 subfamily A member 1; Unc-51 like kinase 3	[8] [13] [16]
18	rs2266782	*FMO3*	Flavin containing monooxygenase 3	[17]
19	rs6015450	*GNAS-EDN3*	GNAS complex locus; Endothelin 3	[8] [13]
20	rs5443	*GNB3*	G protein subunit beta 3	[18]
21	rs17367504	*MTHFR-NPPB*	Methylenetetrahydrofolate reductase; Natriuretic peptide B	[7] [8] [13] [16]
22	rs1799983	*NOS3*	Nitric oxide synthase 3	[19]
23	rs2070744	*NOS3*	Nitric oxide synthase 3	[19]
24	rs3918226	*NOS3*	Nitric oxide synthase 3	[6] [7] [19]
25	rs5068	*NPPA*	Natriuretic peptide A	[20] [21]
26	rs198358	*NPPA-AS1*	NPPA antisense RNA 1	[20]
27	rs1173771	*NPR3-C5orf23*	Natriuretic peptide receptor 3; Chromosome 5 open reading frame 23	[7] [8] [13]
28	rs932764	*PLCE1*	Phospholipase C epsilon 1	[7] [8] [13]
29	rs3754777	*STK39*	Serine/threonine kinase 39	[22]
30	rs13333226	*UMOD*	Uromodulin	[8] [23]

Step 2.

	SNPs included based on assay design	
Number	SNP	Locus
1	rs4341	*ACE*
2	rs4961	*ADD1*
3	rs699	*AGT*
4	rs4762	*AGT*
5	rs5049	*AGT*
6	rs5186	*AGTR1*
7	rs2681472	*ATP2B1*
8	rs1813353	*CACNB2(3')*
9	rs4373814	*CACNB2(5')*
10	rs1799998	*CYP11B2*
11	rs1378942	*CYP1A1-ULK3*
12	rs2266782	*FMO3*
13	rs6015450	*GNAS-EDN3*
14	rs5443	*GNB3*
15	rs17367504	*MTHFR-NPPB*
16	rs1799983	*NOS3*
17	rs2070744	*NOS3*
18	rs3918226	*NOS3*
19	rs5068	*NPPA*
20	rs198358	*NPPA-AS1*
21	rs1173771	*NPR3-C5orf23*
22	rs932764	*PLCE1*
23	rs13333226	*UMOD*

	SNPs excluded during assay design	
Number	SNP	Locus
1	rs4305	*ACE*
2	rs1801253	*ADRB1*
3	rs2004776	*AGT*
4	rs17249754	*ATP2B1*
5	rs4590817	*C10orf107*
6	rs11014166	*CACNB2*
7	rs3754777	*STK39*

Step 3.

	SNPs included in the study (allele frequency comparison, GRS computation)	
Number	SNP	Locus
1	rs4961	*ADD1*
2	rs699	*AGT*
3	rs4762	*AGT*
4	rs5049	*AGT*
5	rs5186	*AGTR1*
6	rs2681472	*ATP2B1*
7	rs1813353	*CACNB2(3')*
8	rs4373814	*CACNB2(5')*
9	rs1378942	*CYP1A1-ULK3*
10	rs2266782	*FMO3*
11	rs6015450	*GNAS-EDN3*
12	rs5443	*GNB3*
13	rs17367504	*MTHFR-NPPB*
14	rs1799983	*NOS3*
15	rs2070744	*NOS3*
16	rs5068	*NPPA*
17	rs198358	*NPPA-AS1*
18	rs1173771	*NPR3-C5orf23*
19	rs932764	*PLCE1*
20	rs13333226	*UMOD*

	SNPs were not recommended		
Number	SNP	Gene	Reason for exclusion
1	rs4341	*ACE*	Did not work. Wrong genotypes called due to poor cluster separation, and too many heterozygots called.
2	rs1799998	*CYP11B2*	Deviation from HWE in our analysis.
3	rs3918226	*NOS3*	Deviation from HWE in our analysis.

Step 4.

	SNPs have effect size estimates publicly available	
Number	SNP	Locus
1	rs4961	*ADD1*
2	rs699	*AGT*
3	rs4762	*AGT*
4	rs5049	*AGT*
5	rs5186	*AGTR1*
6	rs2681472	*ATP2B1*
7	rs1813353	*CACNB2(3')*
8	rs4373814	*CACNB2(5')*
9	rs1378942	*CYP1A1-ULK3*
10	rs6015450	*GNAS-EDN3*
11	rs5443	*GNB3*
12	rs17367504	*MTHFR-NPPB*
13	rs1799983	*NOS3*
14	rs2070744	*NOS3*
15	rs5068	*NPPA*
16	rs198358	*NPPA-AS1*
17	rs1173771	*NPR3-C5orf23*
18	rs932764	*PLCE1*
19	rs13333226	*UMOD*

	SNPs without available effect size	
Number	SNP	Locus
1	rs2266782	*FMO3*

Step 5.

	SNPs included in wGRS analyses	
Number	SNP	Locus
1	rs4961	*ADD1*
2	rs699	*AGT*
3	rs4762	*AGT*
4	rs5049	*AGT*
5	rs5186	*AGTR1*
6	rs2681472	*ATP2B1*
7	rs1813353	*CACNB2(3')*
8	rs4373814	*CACNB2(5')*
9	rs1378942	*CYP1A1-ULK3*
10	rs6015450	*GNAS-EDN3*
11	rs5443	*GNB3*
12	rs17367504	*MTHFR-NPPB*
13	rs1799983	*NOS3*
14	rs2070744	*NOS3*
15	rs5068	*NPPA*
16	rs198358	*NPPA-AS1*
17	rs1173771	*NPR3-C5orf23*
18	rs932764	*PLCE1*
19	rs13333226	*UMOD*

Step 1. On the basis of the systematic literature review altogether 30 SNPs associated with hypertension were identified.

Step 2. During the assay design different SNP pools were created by the service provider. Our decision on the set of SNPs on which the assay was carried out was based on the representation of SNPs with strong (susceptible or protective) influence on the investigated phenotype. In this set suitable for simultaneous genotyping in iPLEX Gold chemistry 23 SNPs were included.

Step 3. In the early stage of genotyping process 1 SNP were not recommended for further analysis by the service provider for the following reason: in case of the rs4341 SNP the assay failed, the observed wrong genotypes called due to poor cluster separation, and too many heterozygotes called. Further 2 SNPs, the 1799998 and rs3918226 deviated from Hardy-Weinberg Equilibrium in the Hungarian general population ($p<0.05$) according to our analyses. The remaining 20 SNPs were involved in comparison of allele frequencies and computation of unweighted GRS.

Step 4. Effect size estimates from association studies were available for 19 SNPs in the literature, consequently only these SNPs could be considered in computation of weighted GRS.

Step 5. Based on the LD pattern none of the pairwise LD of the studied SNPs reached the r^2 threshold of ≥0.8, there were not observed multicollinearity between the polymorphisms, thus it was not necessary to prune SNP from the analysis. Finally, 19 SNPs could be included in the wGRS computation.

Supplementary Table 2. Distribution of study populations by wGRS quintiles (p=0.029)

	Hungarian general population (%)	Hungarian Roma population (%)
1st quintile of wGRS (-0.54 - ≤0.85)	18.51	21.51
2nd quintile of wGRS (0.85 - ≤1.25)	18.77	21.26
3rd quintile of wGRS (1.25 - ≤1.63)	19.54	20.49
4th quintile of wGRS (1.64 - ≤2.08)	21.59	18.37
5th quintile of wGRS (2.09 - ≤4.92)	21.59	18.37

References

1. Johnson, A.D., et al., *Association of hypertension drug target genes with blood pressure and hypertension in 86,588 individuals.* Hypertension, 2011. **57**(5): p. 903-10.
2. Takeuchi, F., et al., *Reevaluation of the association of seven candidate genes with blood pressure and hypertension: a replication study and meta-analysis with a larger sample size.* Hypertens Res, 2012. **35**(8): p. 825-31.
3. Glenn, K.L., et al., *An alternative method for genotyping of the ACE I/D polymorphism.* Mol Biol Rep, 2009. **36**(6): p. 1305-10.
4. Cusi, D., et al., *Polymorphisms of alpha-adducin and salt sensitivity in patients with essential hypertension.* Lancet, 1997. **349**(9062): p. 1353-7.
5. Liu, K., et al., *Alpha-adducin Gly460Trp polymorphism and hypertension risk: a meta-analysis of 22 studies including 14303 cases and 15961 controls.* PLoS One, 2010. **5**(9).
6. Johnson, T., et al., *Blood pressure loci identified with a gene-centric array.* Am J Hum Genet, 2011. **89**(6): p. 688-700.
7. Tragante, V., et al., *Gene-centric meta-analysis in 87,736 individuals of European ancestry identifies multiple blood-pressure-related loci.* Am J Hum Genet, 2014. **94**(3): p. 349-60.
8. Kato, N., et al., *Trans-ancestry genome-wide association study identifies 12 genetic loci influencing blood pressure and implicates a role for DNA methylation.* Nat Genet, 2015. **47**(11): p. 1282-93.
9. Kunz, R., et al., *Association between the angiotensinogen 235T-variant and essential hypertension in whites: a systematic review and methodological appraisal.* Hypertension, 1997. **30**(6): p. 1331-7.
10. Jeunemaitre, X., et al., *Molecular basis of human hypertension: role of angiotensinogen.* Cell, 1992. **71**(1): p. 169-80.
11. Pereira, T.V., et al., *Meta-analysis of the association of 4 angiotensinogen polymorphisms with essential hypertension: a role beyond M235T?* Hypertension, 2008. **51**(3): p. 778-83.
12. Wang, W.Y., R.Y. Zee, and B.J. Morris, *Association of angiotensin II type 1 receptor gene polymorphism with essential hypertension.* Clin Genet, 1997. **51**(1): p. 31-4.
13. International Consortium for Blood Pressure Genome-Wide Association, S., et al., *Genetic variants in novel pathways influence blood pressure and cardiovascular disease risk.* Nature, 2011. **478**(7367): p. 103-9.
14. Levy, D., et al., *Genome-wide association study of blood pressure and hypertension.* Nat Genet, 2009. **41**(6): p. 677-87.
15. Sookoian, S., et al., *Association of the C-344T aldosterone synthase gene variant with essential hypertension: a meta-analysis.* J Hypertens, 2007. **25**(1): p. 5-13.
16. Newton-Cheh, C., et al., *Genome-wide association study identifies eight loci associated with blood pressure.* Nat Genet, 2009. **41**(6): p. 666-76.
17. Bushueva, O., et al., *The Flavin-Containing Monooxygenase 3 Gene and Essential Hypertension: The Joint Effect of Polymorphism E158K and Cigarette Smoking on Disease Susceptibility.* Int J Hypertens, 2014. **2014**: p. 712169.
18. Benjafield, A.V., et al., *G-protein beta3 subunit gene (GNB3) variant in causation of essential hypertension.* Hypertension, 1998. **32**(6): p. 1094-7.
19. Salvi, E., et al., *Genomewide association study using a high-density single nucleotide polymorphism array and case-control design identifies a novel essential hypertension susceptibility locus in the promoter region of endothelial NO synthase.* Hypertension, 2012. **59**(2): p. 248-55.
20. Newton-Cheh, C., et al., *Association of common variants in NPPA and NPPB with circulating natriuretic peptides and blood pressure.* Nat Genet, 2009. **41**(3): p. 348-53.
21. Cannone, V., et al., *The atrial natriuretic peptide genetic variant rs5068 is associated with a favorable cardiometabolic phenotype in a Mediterranean population.* Diabetes Care, 2013. **36**(9): p. 2850-6.
22. Xi, B., et al., *STK39 polymorphism is associated with essential hypertension: a systematic review and meta-analysis.* PLoS One, 2013. **8**(3): p. e59584.
23. Padmanabhan, S., et al., *Genome-wide association study of blood pressure extremes identifies variant near UMOD associated with hypertension.* PLoS Genet, 2010. **6**(10): p. e1001177.
